# Through what mechanisms do protected areas affect environmental and social outcomes?

**DOI:** 10.1098/rstb.2014.0267

**Published:** 2015-11-05

**Authors:** Paul J. Ferraro, Merlin M. Hanauer

**Affiliations:** 1 Carey School of Business and Department of Geography and Environmental Engineering, Whiting School of Engineering, Johns Hopkins University, Baltimore, MD 21218, USA; 2 Economics, Sonoma State University, 1801 E. Cotati Avenue, Stevenson Hall Room 2042, Rohnert Park, CA, USA

**Keywords:** causal mechanisms, parks, reserves, conservation, evaluation, impacts

## Abstract

To develop effective protected area policies, scholars and practitioners must better understand the mechanisms through which protected areas affect social and environmental outcomes. With strong evidence about mechanisms, the key elements of success can be strengthened, and the key elements of failure can be eliminated or repaired. Unfortunately, empirical evidence about these mechanisms is limited, and little guidance for quantifying them exists. This essay assesses what mechanisms have been hypothesized, what empirical evidence exists for their relative contributions and what advances have been made in the past decade for estimating mechanism causal effects from non-experimental data. The essay concludes with a proposed agenda for building an evidence base about protected area mechanisms.

## 1. Introduction

A growing empirical evidence base documents whether, and by how much, protected areas affect the environment and human welfare [1,2]. Documenting these impacts is important, but understanding how exactly protection affects the environment and human welfare is just as important. For example, imagine we have learned that protected areas reduce human exploitation of habitat primarily by preventing road and other infrastructure building. Further imagine we have learned that strict enforcement of resource use bans in protected areas leads to conflict that increases human exploitation of habitat. With a deeper understanding of the mechanisms through which protected areas operate, scholars and practitioners are more likely to design and implement protected areas in ways that enhance their desired impacts and reduce their undesired consequences [3–6].

Empirical support for causal mechanisms also strengthens the credibility of evidence about protected area impacts—if one can demonstrate that the hypothesized mechanisms are operating in ways implicitly assumed in many protected area designs, one can have more confidence in empirical claims about protected area impacts. For example, recent claims that protected areas alleviated poverty or improved conditions in neighbouring communities [7–14] would be more credible if scholars could provide empirical support for plausible mechanisms through which such improvements could happen [15]. For example, one study [6] empirically attributed about two-thirds of its estimated poverty reduction to changes in tourism induced by national parks. Had the study instead found no effects from changes in this mechanism or other oft-proposed mechanisms that the authors also analysed, one might view the estimated reduction in poverty more sceptically—from where could it have come if not the mechanisms analysed in the study? Similarly, claims of no impact of protection on deforestation (e.g. [16]) would be stronger if researchers could demonstrate that the plausible mechanisms are absent or countervailing (e.g. protected area enforcement had little or no effects on the costs of resource exploiters).

Although scholars and practitioners have many (often implicit) hypotheses about mechanisms, they have little empirical support for these hypotheses (see §4). The reason the empirical evidence about mechanisms is scant is less an issue of missing data—although missing data is indeed a problem—and more an issue of misunderstanding what causal mechanisms are and how best to estimate their effects from observable data (as opposed to simulations based on theory, which we do not consider here). These misunderstandings lead scholars to select inappropriate research designs, data and methods to elucidate mechanisms. Without a suitable research strategy, credible conclusions about mechanisms cannot be drawn from empirical studies, and thus the advice that scholars provide to managers may be misguided.

To address these misunderstandings, we differentiate mechanisms from other concepts with which they are commonly conflated in the conservation literature, describe empirical designs that can generate credible evidence about mechanisms and summarize what little is known about the mechanisms by which protected areas may produce effects. In doing so, we aim to make the protected area literature's research terms and designs consistent with the science of causal inference applied in other policy fields.

## 2. What is a mechanism?

**Figure 1.**
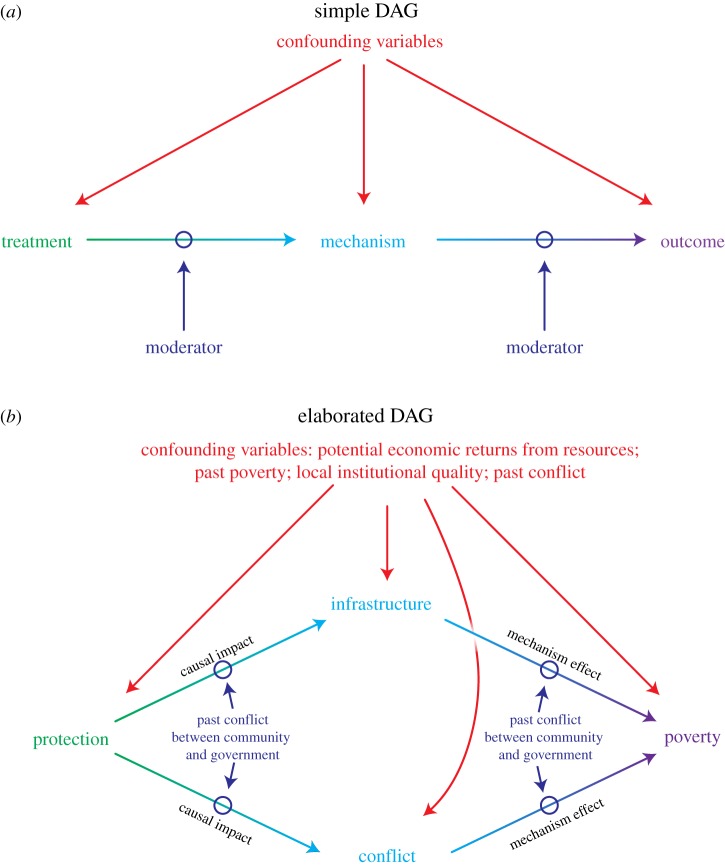
Modified directed acyclic graphs (DAGs) of protected area treatments, moderators and causal mechanisms. Directed arrows indicate direction of causality. (*a*) Simple DAG: a treatment (green) is the form of protection assigned to an area; a moderator (dark blue) is a variable unaffected by the treatment, but which moderates the magnitude of treatment impacts; a mechanism (light blue) is a variable affected by the treatment, which subsequently affects the outcome (purple). Confounding variables (red) jointly affect treatment, mechanisms and outcomes, and may mimic or mask the impacts of treatment. The word mediator is often used in the conservation literature to mean both mechanism and moderator, but differentiating the two concepts is important (see §2*c,d*). (*b*) Elaborated DAG: specific examples of treatments, outcomes, moderators, mechanisms and confounding variables.

In science and practice, one often reads phrases like ‘we identify the factors that determine the impact of protected areas’. Such language fails to differentiate factors that lie on the causal path between the protected areas and the outcomes of interest from factors that are not on the causal path but moderate the effect of protection on outcomes. These differences are illustrated in figure 1 using modified directed acyclic graphs (DAGs).

### (a) Treatments

On the left-hand side of figure 1 is the ‘treatment’, a term borrowed from the experimental sciences and used broadly in the causal inference literature to refer to the causal factor of interest [17]. In the context of protected areas, the treatment describes the form and management of the protected areas being evaluated. In other words, the treatment is the specific intervention or set of activities that is hypothesized to generate environmental or social impacts. For any unit—such as a forest parcel, a coral reef patch or a human community—a treatment can take on two or more values, such as ‘protected’ and ‘unprotected’ (figure 1*b*), or ‘protected with weak enforcement’, ‘protected with moderate enforcement’ and ‘protected with strong enforcement’.

Protected areas thus represent myriad ‘treatment regimens’ that scholars may wish to study, each of which may have different impacts. These regimens vary according to attributes such as legal status (e.g. levels of restrictions ranging from strictly protected to mixed use), *de facto* status (e.g. formal or informal agreements with local communities to allow uses prohibited by law), levels of community participation in management decisions, use of armed guards, levels of penalties for poaching, boundary demarcations, incentive payments, revenue sharing, etc. These attributes of protected areas, such as whether community participation in management is encouraged, are often termed ‘mechanisms' (e.g. [18], p. 65), but they are not mechanisms as defined here.

These attributes of protected areas comprise the treatments (the causes) whose impacts we wish to study—they are not mechanisms through which the treatments operate. Mechanisms are farther along the causal path and explain why, for example, protected areas that share tourism revenues with local communities may have a larger average impact on species abundance than protected areas that do not share revenues. Revenue sharing, which is often confusingly called a ‘financial mechanism’, is an attribute of the treatment. Attributes of the treatment, like revenue sharing, may have been included precisely because they are believed to affect important mechanisms in the desired direction (e.g. revenue sharing with neighbouring communities is believed to favourably affect local perceptions of conservation benefits).

### (b) Outcomes and impacts

On the right-hand side of figure 1 is the ‘outcome’ of interest to scholars or practitioners, which is hypothesized to be causally affected by the treatment; examples include deforestation, ecosystem services, biological diversity and poverty. Each unit that could be assigned a treatment has a potential outcome under each treatment value. If, for example, scholars are interested in the outcome ‘poverty’ and define the treatment by two conditions—‘protected area near the community’ and ‘no protected area near the community’, the potential poverty outcomes are (i) a community's poverty with a protected area nearby and (ii) its poverty without a protected area nearby.

Impacts, or treatment effects, are defined as the difference in potential outcomes under two treatment values. In figure 1*b*, the impact is the difference between poverty when a protected area is nearby and poverty in the same community when a protected area is not nearby. If the treatment were instead defined by the values ‘protected area with the community participating in management decisions' and ‘protected area with no community participation’, the impact is the difference between poverty when the community participates in management decisions and poverty in the same community when it does not participate.

From this perspective, the phrase ‘impacts of protected areas' is too imprecise to be relevant for policy: one must define the two treatment values—the two states of the world—that are being contrasted [19]. For example, one relevant set of impacts may be the impacts from assigning strict protection rather than less strict protection. Another relevant set may be the impacts from assigning strict protection rather than no protection. Each treatment may operate through different mechanisms.

Regardless of the potential treatment values, each treatment effect—each impact of protected areas—should be estimated separately, with the researcher focused on disentangling the causal effect of the treatment from rival confounding factors that are correlated with both the outcomes and where and when different forms of protection are assigned [20]. These confounding factors can mask or mimic the impacts of protected areas. In the context of figure 1*b*, imagine that communities near protected areas have lower poverty, on average, than communities far from protected areas. Does this pattern arise because of protection or because protection is more likely to be assigned near communities with more economic resources? In other words, would communities living near protected areas have had lower poverty even if they had not been living near protected areas? Research designs used to quantify treatment effects (see other articles in this special issue) are similar to, but not the same as, research designs used to quantify mechanism effects (see §3).

### (c) Mechanisms

Treatments affect outcomes through causal mechanisms [17]. These mechanisms lie on a causal path between treatment and outcome (figure 1). Thus, one cannot define mechanisms until one has defined the treatment and the outcome. Mechanisms can also be viewed as intermediate outcomes affected by treatments, or as intermediate treatments (intervening causes) that subsequently have causal effects on outcomes. In the causal pathway, treatment precedes mechanisms, which precede outcome. Each causal path that links protection to an outcome through a mechanism represents a mechanism causal effect (also called an ‘indirect effect’ or ‘natural indirect effect’ [21]).

For example, the mechanism effect of infrastructure on poverty in figure 1*b* is defined as the proportion of the total effect of the treatment that comes from the change in infrastructure induced by the treatment (the upper causal path). If the relevant untreated state is ‘no protection’, the mechanism effect can be viewed as the difference between (i) the total treatment effect and (ii) the treatment effect when the infrastructure value does not change from what it would have been in the absence of protection. In other words, the mechanism effect is the difference in the impact when the mechanism is allowed to change as a result of the treatment compared with the impact when the mechanism is blocked from changing and thus remains at the counterfactual value that it would have taken in the absence of the treatment [6].

A treatment effect can thus fail to materialize because the treatment has no effect on the mechanism, or because the mechanism has no effect on the outcome. For example, Ferraro & Hanauer [6] report that changes in their measures of infrastructure affected poverty in Costa Rica, but they could not detect effects of protected areas on infrastructure (counterfactual condition = no protection). In contrast, they conclude that protected areas affected their measures of forest cover, but changes in forest cover had no detectable effect on poverty.

One could elaborate figure 1*b* even further by specifying, for example, the specific household-level mechanisms through which infrastructure could affect poverty; e.g. better healthcare access (a form of infrastructure) leads to fewer sick days, which increases agricultural productivity, which lowers poverty. How elaborately one wishes to specify the causal paths will depend on (i) the degree to which one has sufficient theory to guide the specification, (ii) the goals of the study (would it be sufficient to know that changes in health clinics caused by protected areas reduced poverty, or does one need to know exactly how such health clinics affected poverty?) and (iii) the data available (can we observe changes in deeper mechanisms, such as changes in agricultural productivity?). Thus, the degree to which we can or should elaborate the causal pathway will depend on context.

**Figure 2.**
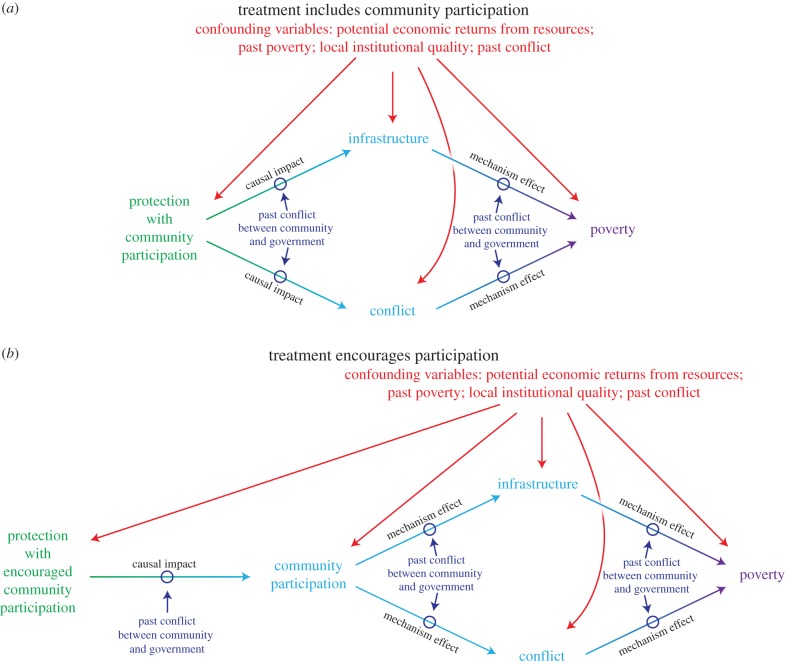
Directed acyclic graphs (DAGs) illustrating distinction between attributes of protected areas and mechanisms. Directed arrows indicate the direction of causality. All causal paths are moderated by past conflict between community and government. (*a*) Treatment is defined as protection in which communities participate in management decisions. Under this treatment definition, levels of participation may vary by protected area and these levels represent different treatment values. The effects of this multi-valued treatment are mediated through changes in infrastructure and conflict. (*b*) Treatment is defined as protection in which community participation in management decisions is encouraged. The level of community participation is not an attribute of protection. Rather, the level of participation induced by the encouragement mediates the effect that protection has on infrastructure and conflict, and thus poverty.

What is a treatment or a mechanism depends on the study. For example, are we interested in estimating the poverty impacts of protected areas in which communities participate in management decisions compared with the counterfactual condition when they do not participate? In this case, participation is not a mechanism—it is part of the treatment (figure 2*a*). Or are we interested in estimating the poverty impacts of protected areas that receive a mandate to encourage community participation compared with the counterfactual condition when they do not receive such a mandate? A mandate for participation does not necessarily mean people participate. People may not participate because, for example, they do not want to participate or because protected area managers do not comply with the mandate. In this case, participation levels are a potential mechanism (figure 2*b*). For example, more people participating may make it more likely that infrastructure is developed in ways that reduce poverty. With 100% compliance with the mandate, the graph in figure 2*b* would look identical to the graph in figure 2*a*. Before we discuss why these definitions matter in practice, we first clarify another relevant concept for protected area impacts.^1^

### (d) Moderators

Moderators are unaffected by the treatment, but influence the magnitude of the treatment effect [22]. Moderators are not on a causal path between treatment and outcome (figure 1). For example, wave exposure has been referred to as a ‘mechanism’ through which coral reef structure is controlled [23], but it is not a mechanism of marine protected area (MPA) impacts; it is a moderator. MPAs do not affect wave exposure, but wave exposure may moderate the degree to which MPAs affect the populations of reef herbivores.

Another way to think of moderators is as ‘context’: protected area impacts may differ across time and space because the context defines which mechanisms can be affected by a treatment and constrains the values that these mechanisms can take. In figure 1*b*, for example, the level of historical (pre-treatment) conflict between a community and the government is hypothesized to moderate the poverty impacts of protected areas. In other words, historical conflict interacts^2^ with protection to generate heterogeneous impacts across time or space. Protection cannot affect *pre*-protection levels of conflict. Past conflicts are not a mechanism. Protection can, however, affect *post*-protection levels of conflict. Thus, post-protection conflict is a possible mechanism through which protected areas affect poverty (e.g. by increasing the likelihood that protected area employees’ fine or imprison local citizens, which can subsequently immiserate households).

A well-known moderator of the effect that protection has on ecological and social indicators is the economic potential of the resources [24]. For example, terrestrial protected areas may be more likely to exacerbate poverty in neighbouring communities when they are assigned to productive agricultural lands because the foregone agricultural benefits from protection in such contexts are high [25–27]. Economic potential is an example of a moderator that is also a confounding variable (figure 1): these variables affect treatment assignment, mechanism values and outcome values. They thus can also moderate impacts by influencing the form of treatments that are assigned across time and space. For example, strictly protected areas have been shown in some countries to be more likely to be assigned to areas of lower economic potential than are mixed-use protected areas, thereby limiting how much avoided deforestation strict protection can generate [19,28].

### (e) Why do definitions matter?

To select credible empirical designs, collect appropriate data and apply the best methods, scientists must clearly differentiate moderators, mechanisms and treatments. These differences, however, are often not clear in published studies. For example, outside the context of protected areas, an important study by Persha *et al.* [29] develops a unique, broad dataset to shed light on forest management and its effects on tree species richness and subsistence livelihoods in six nations. The authors regress a joint measure of species richness and livelihoods on forest size, the extent of rulemaking participation by local communities and dependence on the forest for extractive commercial livelihoods. Based on the estimated coefficients, they conclude that ‘forest systems are more likely to have sustainable outcomes (above-average tree species richness and subsistence livelihoods) when local forest users participate in forest rulemaking … The size of the forest and the extent to which the forest provided commercial livelihoods to households are also important factors associated with synergies across social and ecological outcomes’ ([29], pp. 1606–1607).

The authors refer to the three variables (forest size, participation and commercial livelihoods) as ‘mediating variables’, but the treatment (cause) that the variables mediate is not clear. In our view, the language used in the text implies that participation is a treatment attribute (forest management with and without participation), forest size is a moderator and provision of commercial livelihoods is a mechanism. If our interpretations are correct (and we are not claiming they are), the authors' empirical design yields important hypotheses for future tests, but does not yield clear conclusions about treatment effects (impacts), mechanism effects or moderating effects; i.e. the estimated regression coefficients cannot be easily interpreted as any of these effects and thus have unclear implications for conservation in practice. For example, unless one is willing to make the strong (and not very credible) assumption that there are no systematic differences between communities that participate in forest rulemaking and those that do not, one cannot use the study results to draw the conclusion that forests are more likely to have sustainable outcomes when local forest users participate in forest rulemaking. Likewise, unless one is willing to assume that observed differences in the extent of commercial livelihoods across communities are entirely a result of participation, one cannot draw the conclusion that participation improves outcomes through the mechanism of commercial livelihoods.

Different empirical designs are needed to estimate the effects of treatments, mechanisms and moderators. Specifically, different combinations of data, untestable assumptions and methods are needed to draw conclusions with causal interpretations.^3^ In conservation science, one often sees treatments, mechanisms and moderators conflated and thus the evidence base is weaker than it might otherwise be.

## 3. Empirical estimation of mechanism effects

Conservation researchers are beginning to realize that the first step in estimating the effect of protected areas on environmental and social outcomes is *not* to gather data or to choose designs or methods. Rather, the first step is to characterize the process through which some units (e.g. species, habitats, humans) are exposed to protected areas or their attributes, and other units are not [20]. Why, for example, are some areas assigned strict protection that curtail most human uses of the resources, while other areas allow mixed uses and other areas are left unprotected? Why are some protected areas sharing revenues with local communities and others are not? The answers to these questions point to potentially confounding variables (figure 1), as well as creative ways to identify the effect of the protected areas and their attributes (e.g. instrumental variables; see [20]). In the absence of a solid understanding of ‘selection into treatment’, credible causal inferences are simply not possible. An understanding of the selection process guides the choice of design, data and methods (see box 1 for a common misunderstanding in the conservation science literature).

Box 1. **Studies that control for mechanisms, rather than estimate mechanism effects.**

To estimate the impacts, one aims to identify and eliminate the influence of confounding factors that systematically affect both the outcomes and the treatments. For example, terrestrial protected areas are often located where agricultural potential is limited. Thus, impact studies that compare outcomes between protected and unprotected parcels need to compare parcels of similar agricultural potential (i.e. control for, or condition on, agricultural potential). The lack of pre-protection measures of these confounding factors is a widespread problem in conservation science. Scientists are often forced to use post-protection measures [30–36]. If, however, the confounding factors are themselves mechanisms, studies that control for their post-protection values are inadvertently blocking the effects of protected areas that occurred through these mechanisms. For example, studies often attempt to control for access to roads. When pre-protection road data are unavailable, authors condition on post-protection road data. If protected areas reduce habitat conversion by retarding road network growth, the authors have eliminated this mechanism effect from their analysis, thereby underestimating the absolute magnitude of protected areas' impacts (the same problem arises when studies condition on post-treatment outcomes, like fish counts or forest cover). Introducing this form of bias may be unavoidable when, post-protection, a factor is a mechanism and, pre-protection, it is a confounding factor whose values cannot be observed by the analyst. However, the dilemma should be acknowledged and the implications for the conclusions that can be drawn should be made explicit.

The same deep thinking about selection is also required to estimate mechanism effects. In addition to characterizing how units are selected for protection, researchers must characterize why some units are exposed to particular values of a mechanism and others are exposed to other values. Guided by this characterization, researchers can try to isolate changes in the mechanisms that come from protected areas, rather than from other causes that may also affect the mechanisms and outcomes (i.e. confounding variables). For example, protected areas are hypothesized to affect environmental and social outcomes through their effects on infrastructure quantity and quality. However, infrastructure quantity and quality are affected by a variety of other factors that also affect environmental and social outcomes; for example, local economic growth. One must control for the influence of these other factors in order to isolate the effect of infrastructure that comes only from the establishment of protected areas (the mechanism effect).

Ideally a researcher would have control over the values of the treatment and mechanisms. The advantage afforded by such control is best illustrated through a thought experiment. For example, to estimate the impact of terrestrial protection on health through changes in infrastructure, a researcher would run two sequential experiments. In the first experiment, protection is randomly assigned among a pool of eligible candidate areas, after which the average impact of protection on health in neighbouring communities is estimated. The second experiment starts with a clean slate and assigns protection to identical areas. In this second experiment, however, the effect of protection on infrastructure is blocked; i.e. infrastructure is held at the same level that would be observed if there were no protected area. In this experiment, the health levels in protected communities include all of the effects of protection except those that arise from changes in infrastructure. The difference in the average impacts in the first and second experiments represents the effect of protection on health that works through protection's effect on infrastructure: the mechanism effect.

Such ‘time-travel’ experiments are not feasible. Therefore, to isolate mechanism effects, researchers need a clear, elaborate theory of the plausible causal pathways and the potential confounders. This elaborate theory points to appropriate empirical designs, which point to appropriate data, which point to appropriate methods to draw inferences from the data.

### (a) Designs

To estimate mechanism causal effects, researchers can use one of three empirical designs. The most direct, and typically least feasible, design is one in which a mechanism is experimentally manipulated [37]. For example, to test the importance of tourism business opportunities as a mechanism affecting poverty around protected areas, one could randomly assign government support for tourism infrastructure and associated marketing across protected areas.^4^ To test the importance of expected penalties for illegal use of protected areas in the benefit–cost calculation of users, one could experimentally manipulate penalties or enforcement effort across protected areas. With an experimental approach, some of the variation in the mechanism values are controlled by the experimenter. Thus mechanism experiments often have high internal validity (i.e. the degree to which rival explanations for the observed correlations between mechanism and outcome can be ruled out). Nevertheless, mechanisms may be manipulated in ways that are not typical of protected areas and thus the experiments could have low external validity.

A second approach is to combine non-experimental (observational) data, statistical methods, and an elaborated causal model that rests on strong assumptions about treatment and mechanism assignment (i.e. strong assumptions about how different units came to be exposed to different values of the treatment *and* the mechanisms). For example, Ferraro & Hanauer [6] assume that after conditioning on six confounding variables, the treatment (protection) and mechanisms (tourism business opportunities, infrastructure and land cover change) are ‘as-if’ randomly assigned. They then use a two-step estimator that combines matching and regression methods to eliminate these confounding variables as rival explanations. They first estimate the effect of protected areas on the mechanisms (step 1), and then estimate the effect of the mechanisms on poverty (step 2). A variety of potential methods can be used (e.g. structural equation modelling), but all approaches require at least one of the two causal paths (treatment to mechanism, or mechanism to outcome) be modelled using a specification based on theory and expert opinion. Non-experimental designs like these also tend to assume that the mechanisms are isolated – in other words, in addition to no links from confounding variables to the mechanisms, there are no links between the mechanisms themselves (no directed arrows between mechanisms in figure 1*b*).

When the assumptions underlying these non-experimental designs are untenable, researchers can try a ‘partial identification’ approach that attempts to use weaker, but more plausible assumptions, to place upper and lower bounds on the mechanism effects (examples of partial identification to estimate conservation treatment effects include [38,39]). Sensitivity tests to hidden bias can also be used to consider how large a deviation from the underlying assumptions would be required to change the conclusions in a scientifically or policy-relevant manner [40].

In the absence of a credible experimental or non-experimental design, one can attack the problem indirectly using theory and supporting data. For example, to assess whether tourism is a mechanism through which Costa Rica's protected areas have affected human welfare, Robalino & Villalobos [41] apply a test of known effect [42]. The authors posit that if the tourism induced by protected areas improved local wages, then impacts should be most pronounced in communities nearer park entrances, where tourism activities are most concentrated. The authors indeed detect a pattern in which wages increase with proximity to a park entrance, and conclude that the evidence favours the hypothesized mechanism. For an excellent example outside of protected areas, see Alix-Garcia *et al.* [43], who combine results from a pilot randomized controlled trial and a test of known effect (based on economic theory) to generate evidence about the mechanisms through which an anti-poverty programme affected deforestation in Mexico.

## 4. Theory and evidence about mechanisms: environmental outcomes

### (a) Theory

Elaborate, mechanism-based theories, grounded in the natural and social sciences, are absolutely essential to guide empirical analyses. Unfortunately, we know of no publications that have clearly described plausible mechanisms through which protected areas have impacts on environmental outcomes. Nevertheless, based on our understanding of the debates over protected area design and the popular elements of protected area treatments, we propose a suite of plausible mechanisms through which protected areas can affect ecological outcomes such as species richness, species abundance and levels of ecosystem services, or land- or sea-use outcomes such as poaching, deforestation, fires and reef dynamiting (which are themselves mechanisms through which ecological outcomes such as species abundance are affected by protection).

**Figure 3.**
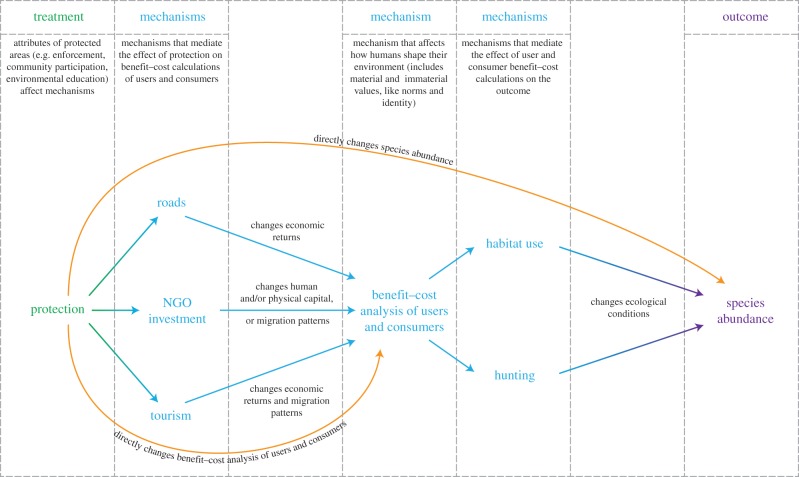
Simple directed acyclic graph (DAG) of protected area mechanisms that mediate protection's impact on species abundance. Directed arrows indicate the direction of causality. On top of directed arrows are descriptions of intermediary mechanisms that are left implicit in the DAG. The DAG ignores moderators of impact and potential confounders that may affect both the mechanisms and outcomes. These confounders should be made explicit in any effort to estimate the sign and magnitudes of the mechanism effects.

No single model or graph can describe causal pathways for all contexts. Figure 3 presents a specific example of a causal DAG: the effect of protected areas (compared to no protection) on species abundance. Some of the mechanisms in the DAG are typically observable (e.g. roads), while others may be observed with substantial error (e.g. tourism), or be unobservable (e.g. benefit–cost calculations). Depending on the context or the objective, the DAG could be elaborated by further differentiating mechanisms (e.g. road density versus road quality) or by adding mechanisms (e.g. the implied ecological mechanisms that mediate the effects of changes in human behaviours on species abundance).

The primary mechanism through which protected areas can affect environmental outcomes is through their effect on human decisions to use or consume resources. In other words, protected areas affect the benefit–cost calculation of potential users and consumers of the protected resources and related ecosystems. The benefits and costs may be tangible (e.g. cash from tourism revenues; fines for poaching; foregone agricultural production) or intangible (e.g. psychic costs from conflict over contested resources; loss of cultural identity).

The benefit–cost calculations can be affected by aspects of the treatment – in other words, the specific attributes of the protected areas (bottom directed arrow in figure 3). For example, changes in benefit–cost calculations can be achieved through the protected areas' application of enforcement, which affects user and consumer perceptions of the severity of the penalty, the probability of detection, and the probability of penalty assignment given detection. Changes in benefit–cost calculations can also be achieved through other protected area management attributes. For example, allowing neighbouring communities to play a role in making management decisions or share in the tourism revenues may offer new benefits of protection to communities, create new institutions, establish incentives for reciprocity, increase access security to resources, or reduce the perceived psychic costs related to the government or other outside groups making property rights claims over local resources. Changes in benefit–cost calculations can also be achieved through information transfer, such as conservation education, moral suasion and public shaming (often of private firms). These attributes can create new preferences and norms that favour ecosystem protection (e.g. [44]). They can also reduce the psychic costs that arise from conflict over contested resources.

Consumer and user benefit–cost calculations can be affected by changes in other mechanisms that protection affects (middle three directed arrows in figure 3); in other words, a mechanism that precedes the mechanism of the benefit–cost calculation. For example, protected areas may directly enhance or retard the growth in road networks or processing plants in a region, which then affects the economic returns from exploiting the resources. Protected areas may induce non-governmental organizations to invest in local physical and human capital that changes how residents use resources or attracts new residents, who subsequently affect supply and demand. Protected areas may induce an influx of tourists to a region, which may raise demand for resource extraction or may expose local residents to alternative livelihoods (or ideas). The same tourists could induce market changes in health clinics or communication services, which subsequently affect the relative returns of different economic activities or affect immigration and emigration. Protected areas that encourage community participation may benefit from increased local monitoring that may increase the productivity of government enforcement (e.g. local people notify guards of poachers operating in the park).

The effects of changes in benefit–cost calculations are mediated by other mechanisms related to human uses of resources (in figure 3, the mechanisms that follow the benefit–cost calculation mechanism). Changes in resource use may affect outcomes through their effects on ecological mechanisms, such as larval dispersal or species–area relationships.^5^ They may also be mediated by a household welfare mechanism (not in figure 3). For example, legal restrictions may reduce the returns to using traditional resources (first mechanism), which may make poor households consume less of these resources (second mechanism), which in turn may make the households poorer (third mechanism) and which may lead them to decide that defiance through increased poaching or wild fires is an effective strategy to improve their welfare over time (fourth mechanism, which subsequently affects an outcome such as species abundance).

A mechanism in one study may be an outcome in another study. For example, we often cannot observe ecological outcomes, and thus restrict ourselves to studying protected area impacts on human behaviours such as deforestation or fishing. However, in a study that uses ecological indicators such as species abundance as the outcome, the human behaviours are mechanisms through which protection affects the ecological indicator.

In studies that use ecological indicators, rather than human behaviours, as outcomes, the treatments may affect the outcome only through ecological mechanisms (top directed arrow of figure 3). For example, ecological indicators may be affected by protected area manager actions, such as revegetation or removal of invasive species. For species outcomes, protected areas must affect the key attributes of the environment on which a particular species depends, which sometimes may be outside the protected area (i.e. the protected area configuration and enforcement actions, which are part of the treatment, are important).

### (b) Empirical evidence

Given that the plausible mechanisms through which protected areas may affect environmental outcomes have not been carefully elaborated in the scientific literature, it may not be surprising that the empirical evidence for the presence and magnitude of mechanism effects is scant. To our knowledge, no studies have attempted to estimate mechanism effects using any of the designs described in §4*a* (claims about plausible ecological mechanisms have been made, but we could find no studies that estimate the mechanism effects that arise from protected area treatments).

## 5. Theory and evidence on mechanisms: social outcomes

### (a) Theory

The impacts of protected areas on social outcomes (human welfare) have been the focus of extensive debate in the past decades (e.g. [46]). Although a variety of elaborated pathways have been discussed (e.g. [6,10,11,47,48]), two broad mechanism themes are most frequently cited in the debate: (i) protected areas restrict access to resources, which reduces human welfare, and (ii) protected areas bolster ecosystem services, which increases human welfare. These mechanism themes are linked to the protected area treatments, the benefit–cost calculations of users and consumers and myriad ecological mechanisms that shape biodiversity and ecosystem services.

Thus the most plausible mechanisms for social outcomes are similar to the most plausible mechanisms for environmental outcomes. For example, in figure 3, tourism (first mechanism) can affect the benefit–cost analysis of users (second mechanism) by offering alternative employment opportunities. These alternative employment opportunities will in turn have an effect on wages, incomes and work satisfaction (a set of third mechanisms), which will in turn affect a variety of outcomes, such as health, education and material consumption. As in the studies of environmental impacts of protected areas, a mechanism in one study of the social impacts of protected areas may be an outcome in another study. Moreover, a single DAG (causal model or graph) is unlikely to describe causal pathways for all treatments or contexts.

In §4*a*, we noted that human welfare can be a mechanism through which protected areas affect environmental outcomes. Similarly, environmental outcomes (e.g. land or sea use, species abundance and ecosystem services) can be mechanisms through which protected areas affect human welfare (for a review of the hypothesized linkages between ecosystem services and human welfare, see [49]). For example, Ferraro & Hanauer [6] posit that changes in forest cover induced by protected areas in Costa Rica can lead to a variety of mechanism effects on poverty by changing agricultural production and the supply of desirable (e.g. pollination) and undesirable (e.g. crop predators) ecosystem services.

As noted earlier, the degree to which we can or should elaborate the causal pathway will depend on context. For example, Ferraro & Hanauer's [6] analysis only measures the total mechanism effect from changes in forest cover. They cannot identify the mechanism effects from agriculture separate from the mechanism effects of ecosystem services because they cannot observe these two deeper mechanisms. Because ecosystem services are typically not directly observable, disentangling the mechanisms associated with resource use changes that subsequently affect social outcomes may be difficult without the help of modellers [50].

### (b) Evidence

To our knowledge, no studies have used an experimental design to explore mechanism effects of protected areas on social outcomes. We know of only one study that estimates mechanism effects by combining data, strong assumptions and statistical methods to control for confounding factors that affect treatment, mechanisms and outcomes. Ferraro & Hanauer [6] attempt to estimate mechanism effects of protected areas in Costa Rica that arise from changes in tourism, infrastructure (roads, schools, health clinics) and land cover. Their results imply that two-thirds of the estimated poverty alleviation impact arose from protected areas' effects on tourism activity and the other third from protected areas' effects on unmeasured attributes of their mechanisms or other mechanisms. No mechanism effects from changes in infrastructure or forest cover were detected. Their measures of the mechanisms, however, were crude [4]. For example, tourism activity is multi-dimensional; in contrast, the tourism mechanism in this study was unidimensional because historical multi-dimensional data on tourism activities do not exist. Thus, even if the variable they use isolates only the tourism mechanism, the authors cannot say what exactly about tourism helps to reduce poverty.

The Ferraro and Hanauer study also makes clear the strong assumptions required to study mechanisms when no measures of the mechanisms exist. In their estimate of the tourism mechanism effect, they do not use a measure of the mechanism, but rather an attribute of the treatment. They measure whether a protected area has a formal entrance through which visitors can enter (some parks do and some do not). They then make an untested, but plausible, assumption that tourists do not arrive in economically relevant numbers in the absence of an entrance. With this assumption, the treatment attribute serves as a surrogate (proxy) for the mechanism, allowing the authors to estimate the mechanism effect.

A few studies make claims about mechanism effects based on indirect approaches using theory and supporting data. The Robalino & Villalobos [41] study that attempts to uncover evidence in favour of a tourism mechanism effect on incomes in neighbouring communities was discussed in §3*a*. Using data from Cambodia and strong assumptions to identify treatment effects, Clements *et al.* [14] report that protected areas with substantial eco-market payment opportunities to local residents had larger well-being impacts than protected areas with smaller payments. They conclude that eco-market opportunities induced by protected areas can improve the welfare of neighbouring communities. McNally *et al.* [10] find that Saadani National Park in Tanzania reduced mangrove deforestation and that mangrove area is positively correlated with income from fishing and shrimping. Based on these correlations, they argue that protection of mangroves increased welfare by bolstering ecosystem services. Baird [51] uses quantitative and qualitative data to support the hypothesis that protected areas improve household welfare by allowing local communities to procure financial support from a greater diversity of external organizations. Canavire-Bacarreza & Hanauer [11] incorporate expert opinion into an evaluation of Bolivia's protected areas to argue that protected areas decreased poverty, at least in part, by increasing community cohesion.

## 6. Conclusion

Without an understanding of the mechanisms through which protected areas operate, decision-makers will find it difficult to design effective protected area networks. Effective networks cannot be designed without evidence about which mechanisms are most important and how we can best influence them. To date, however, the evidence base is weak because our theories about mechanisms tend to be imprecise and our empirical designs for estimating mechanism effects tend to be inappropriate. To advance our understanding, we propose a three-point agenda for scholarship and practice.

First, scholars and practitioners need to better understand what mechanisms and mechanism effects are (and are not). Without clearer differentiation of treatments, moderators and mechanisms in the context of protected areas research, scholars and practitioners will be constrained in their ability to develop theory, collect data, and draw inferences from data about the mechanisms through which protected areas operate.

Second, with a better conceptual understanding of mechanisms, scholars and practitioners need to develop better theory about protected area mechanisms. The plausible mechanisms, their confounders, their moderators and any potential interactions among mechanisms need to be more explicitly elaborated to better guide data collection and empirical research.

Third, with better theory, scholars and practitioners need to apply more appropriate empirical designs for generating credible evidence about protected area mechanisms. Better theory will be most critical when mechanisms (or their effects on outcomes) cannot be observed directly and we must depend on indirect approaches, such as tests of known effects, to generate credible evidence about mechanisms. Theories derive power from their ability to exclude explanations. Thus attempts to draw conclusions about mechanisms based on theoretical predictions of empirical patterns require powerful theories.

When possible, scholar–practitioner collaborations should seek to experimentally manipulate treatments and mechanisms [52]. When experimental manipulation is not possible, we should apply empirical designs that are capable of credibly estimating mechanism effects separate from rival explanations that could also explain the observed patterns in the data. Such non-experimental designs will rest on strong, untestable assumptions. Thus when using these designs, we must make very clear why we believe that unobserved variables that affect the mechanisms are not systematically correlated with the outcomes or where and when protected areas are established (such confounders could include unobserved mechanisms). In cases where such strong assumptions are implausible, we should explore the implications of weakening the assumptions. Under weak, but much more plausible assumptions, identifying policy-relevant bounds on mechanism effects may still be possible.

Generating persuasive evidence about protected area mechanisms will entail drawing on numerous studies and continuing to apply a healthy dose of scepticism to the evidence offered. Persuasive evidence about mechanisms is difficult to develop even in the best of circumstances (e.g. cases where the researcher can randomly assign both treatment and mechanism; [35,36]). Protected areas do not represent the best of circumstances. Thus developing strong evidence about mechanisms will be much harder. This challenge should not cause us to shy away from much-needed research on mechanisms or from drawing on even limited evidence to improve conservation practice. It does, however, make clear that conservation evidence will remain limited until scholars and practitioners make substantial advances in their scientific efforts to understand protected area impacts.

## Endnotes

1 Readers with some training in impact evaluation may like to think of figure 2*a* as representing a study seeking to estimate the average treatment effect on the treated and the average mechanism effects on the treated (where treatment is protection plus participation). Figure 2*b* represents a study seeking to estimate the average intent to treat effect and the average mechanism intent to treat effects (in other words, the causal effects of treatment *assignment* rather than of treatment itself).

2 We do not mean ‘interact’ in the sense of two variables having separate independent causal effects and an additional joint causal effect. The moderator may have no independent causal effect.

3 In the Persha *et al.* [29] case, for example, one could estimate the treatment effects of participation and the mechanism effects of commercial livelihoods through designs that control for confounding factors that affect both which communities participate in rulemaking or commercial activities and the species richness or subsistence livelihoods. To estimate the moderating effects of forest size, one could interact forest size with different values of the treatment variable (instead of estimating marginal effects at different values of forest size, as was done in the study).

4 To draw inferences about the mechanism effect, however, one must either assume that support for tourism only affects poverty through its effect on tourism business opportunities, or control for other pathways from support to poverty. Also, unlike an experiment where the business opportunities themselves are randomized, an experiment that randomizes government support may not have an effect on tourism business opportunities in all units (i.e. it is a randomized encouragement design that, without further assumptions, only identifies a local average treatment effect on units whose mechanism values are affected by the encouragement).

5 Resource uses and ecological processes outside protected area boundaries play a role in protected area impacts [45], but unless they are affected by the protected areas, they are moderators, not mechanisms.
